# Methyl 2-hydr­oxy-3-nitro­benzoate

**DOI:** 10.1107/S1600536809024301

**Published:** 2009-06-27

**Authors:** Yan-Zhu Liu, Yong-Xiu Li, Ling Zhang, Xia Li

**Affiliations:** a Department of Chemistry, Nanchang University, Nanchang 330031, People’s Republic of China

## Abstract

The title compound, C_8_H_7_NO_5_, assumes an approximately planar mol­ecular structure with an intra­molecular O—H⋯O hydrogen bond between the hydr­oxy and carboxyl­ate groups. Weak inter­molecular C—H⋯O hydrogen bonding is present in the crystal structure.

## Related literature

For the properties of 2-hydroxy­benzoyl compounds, see: Konopacka *et al.* (2005 ▶); Sonar *et al.* (2007 ▶); Willian & Layne (2001 ▶); Huang *et al.* (1996 ▶). For bond-length data, see: Allen *et al.* (1987 ▶).
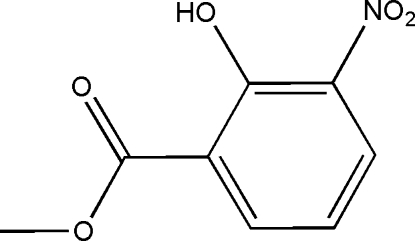


## Experimental

### 

#### Crystal data

C_8_H_7_NO_5_
*M* *_r_* = 197.15
Monoclinic, 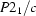

*a* = 7.6120 (10) Å
*b* = 11.716 (2) Å
*c* = 9.656 (2) Å
β = 101.830 (10)°
*V* = 842.9 (3) Å^3^
*Z* = 4
Mo *K*α radiation
μ = 0.13 mm^−1^
*T* = 291 K
0.30 × 0.20 × 0.20 mm

#### Data collection

Bruker SMART CCD area-detector diffractometer
Absorption correction: none
4045 measured reflections
1473 independent reflections
965 reflections with *I* > 2σ(*I*)
*R*_int_ = 0.046

#### Refinement

*R*[*F* ^2^ > 2σ(*F* ^2^)] = 0.055
*wR*(*F* ^2^) = 0.110
*S* = 1.02
1655 reflections
129 parameters
H-atom parameters constrained
Δρ_max_ = 0.48 e Å^−3^
Δρ_min_ = −0.40 e Å^−3^

### 

Data collection: *SMART* (Bruker, 2000 ▶); cell refinement: *SAINT* (Bruker, 2000 ▶); data reduction: *SAINT*; program(s) used to solve structure: *SHELXS97* (Sheldrick, 2008 ▶); program(s) used to refine structure: *SHELXL97* (Sheldrick, 2008 ▶); molecular graphics: *ORTEP-3 for Windows* (Farrugia, 1997 ▶); software used to prepare material for publication: *WinGX* (Farrugia, 1999 ▶).

## Supplementary Material

Crystal structure: contains datablocks I, global. DOI: 10.1107/S1600536809024301/xu2532sup1.cif

Structure factors: contains datablocks I. DOI: 10.1107/S1600536809024301/xu2532Isup2.hkl

Additional supplementary materials:  crystallographic information; 3D view; checkCIF report

## Figures and Tables

**Table 1:** Hydrogen-bond geometry (Å, °)

*D*—H⋯*A*	*D*—H	H⋯*A*	*D*⋯*A*	*D*—H⋯*A*
O1—H1*A*⋯O4	0.96	1.70	2.554 (2)	146
C4—H4*A*⋯O2^i^	0.93	2.57	3.321 (3)	138
C6—H6*A*⋯O4^ii^	0.93	2.49	3.336 (3)	151
C8—H8*B*⋯O1^ii^	0.96	2.59	3.305 (3)	131

Symmetry codes: (i) 
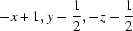
; (ii) 
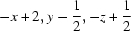
.

**Fig. 1.**
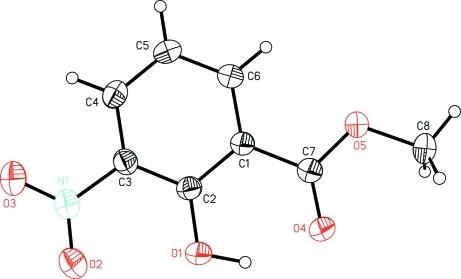
The molecular structure of the title compound with displacement ellipsoids at the 30% probability level. The dashed line indicates hydrogen bonding.

## supplementary crystallographic information

### Comment

Methyl salicylate and its analogues are useful intermediates in organic synthesis and show potential applications for functional materials and drugs (Konopacka *et al.*, 2005; Sonar *et al.*, 2007; Willian & Layne, 2001; Huang *et al.*, 1996). In this paper, the structure of the title compound is reported.

The molecular structure of (I) is shown in Fig. 1. The bond lengths and angles are within normal ranges (Allen *et al.*, 1987). There is an intramolecular hydrogen bond between the hydroxy group and the carboxyl group, and the whole molecule is planar except for the methyl H atoms. The crystal structure is stabilized by weak intermolecular C—H···O hydrogen bonding (Table 1).

### Experimental

The methyl salicylate (3 ml) and Fe(NO_3_)_3_.9(H_2_O) (3 g) were dissolved in ethyl acetate (50 ml), and the solution was refluxed for 1 h. The resulting mixture was cooled and filtered. The yellow single crystals were obtained from the filtrate by slowly evaporating ethyl acetate.

### Refinement

H atoms were located geometrically and treated as riding atoms with C—H = 0.93 (aromatic), 0.96 Å (methyl) and O—H = 0.96 Å, and with *U*_iso_(H) = 1.2*U*_eq_(C) for aromatic H atoms and 1.5*U*_eq_(C,*O*) for the others.

### Crystal data

**Table:**

C_8_H_7_NO_5_	*F*(000) = 408
*M**_r_* = 197.15	*D*_x_ = 1.554 Mg m^−^^3^
Monoclinic, *P*2_1_/*c*	Mo *K*α radiation, λ = 0.71073 Å
Hall symbol: -P 2ybc	Cell parameters from 1211 reflections
*a* = 7.612 (1) Å	θ = 2.7–22.6°
*b* = 11.716 (2) Å	µ = 0.13 mm^−^^1^
*c* = 9.656 (2) Å	*T* = 291 K
β = 101.83 (1)°	Block, yellow
*V* = 842.9 (3) Å^3^	0.30 × 0.20 × 0.20 mm
*Z* = 4	

### Data collection

**Table:**

Bruker SMART CCD area-detector diffractometer	965 reflections with *I* > 2σ(*I*)
Radiation source: fine-focus sealed tube	*R*_int_ = 0.046
graphite	θ_max_ = 26.0°, θ_min_ = 2.7°
φ and ω scans	*h* = −8→9
4045 measured reflections	*k* = −13→13
1473 independent reflections	*l* = −11→6

### Refinement

**Table:**

Refinement on *F*^2^	Secondary atom site location: difference Fourier map
Least-squares matrix: full	Hydrogen site location: inferred from neighbouring sites
*R*[*F*^2^ > 2σ(*F*^2^)] = 0.055	H-atom parameters constrained
*wR*(*F*^2^) = 0.110	*w* = 1/[σ^2^(*F*_o_^2^) + (0.02*P*)^2^ + 0.55*P*] where *P* = (*F*_o_^2^ + 2*F*_c_^2^)/3
*S* = 1.02	(Δ/σ)_max_ < 0.001
1655 reflections	Δρ_max_ = 0.48 e Å^−^^3^
129 parameters	Δρ_min_ = −0.40 e Å^−^^3^
0 restraints	Extinction correction: *SHELXL97* (Sheldrick, 2008), Fc^*^=kFc[1+0.001xFc^2^λ^3^/sin(2θ)]^-1/4^
Primary atom site location: structure-invariant direct methods	Extinction coefficient: 0.010 (3)

### Special details

**Table:**

Experimental. ^1^H-NMR (CDCl_3_, 500 MHz): δ4.03 (s, 3 H), 7.20(s, 1 H), 8.15-8.19 (d, 2 H), 12.02 (s, 1 H).
Geometry. All e.s.d.'s (except the e.s.d. in the dihedral angle between two l.s. planes) are estimated using the full covariance matrix. The cell e.s.d.'s are taken into account individually in the estimation of e.s.d.'s in distances, angles and torsion angles; correlations between e.s.d.'s in cell parameters are only used when they are defined by crystal symmetry. An approximate (isotropic) treatment of cell e.s.d.'s is used for estimating e.s.d.'s involving l.s. planes.
Refinement. Refinement of *F*^2^ against ALL reflections. The weighted *R*-factor *wR* and goodness of fit *S* are based on *F*^2^, conventional *R*-factors *R* are based on *F*, with *F* set to zero for negative *F*^2^. The threshold expression of *F*^2^ > σ(*F*^2^) is used only for calculating *R*-factors(gt) *etc*. and is not relevant to the choice of reflections for refinement. *R*-factors based on *F*^2^ are statistically about twice as large as those based on *F*, and *R*- factors based on ALL data will be even larger.

### Fractional atomic coordinates and isotropic or equivalent isotropic displacement parameters (Å^2^)

**Table:**

	*x*	*y*	*z*	*U*_iso_*/*U*_eq_	
C3	0.6545 (3)	0.9162 (2)	−0.0890 (2)	0.0465 (6)	
C4	0.6080 (3)	0.8030 (2)	−0.1125 (3)	0.0541 (7)	
H4A	0.5248	0.7822	−0.1931	0.065*	
C5	0.6836 (4)	0.7212 (2)	−0.0176 (3)	0.0581 (8)	
H5A	0.6523	0.6448	−0.0337	0.070*	
C6	0.8059 (3)	0.7525 (2)	0.1011 (3)	0.0504 (7)	
H6A	0.8577	0.6966	0.1649	0.060*	
C1	0.8541 (3)	0.8654 (2)	0.1281 (2)	0.0433 (6)	
C2	0.7769 (3)	0.9507 (2)	0.0320 (2)	0.0449 (6)	
C7	0.9867 (3)	0.8997 (2)	0.2543 (3)	0.0499 (7)	
C8	1.1859 (4)	0.8424 (2)	0.4632 (3)	0.0654 (9)	
H8C	1.2856	0.8819	0.4384	0.098*	
H8B	1.2277	0.7740	0.5140	0.098*	
H8A	1.1297	0.8907	0.5217	0.098*	
N1	0.5694 (4)	0.9981 (2)	−0.1954 (3)	0.0726 (8)	
O2	0.6026 (3)	1.09798 (19)	−0.1803 (2)	0.0830 (7)	
O3	0.4677 (4)	0.96270 (19)	−0.2983 (2)	0.1045 (9)	
O1	0.8187 (3)	1.06068 (13)	0.05425 (19)	0.0651 (6)	
H1A	0.9073	1.0676	0.1402	0.098*	
O4	1.0286 (3)	0.99828 (15)	0.2831 (2)	0.0692 (6)	
O5	1.0566 (2)	0.81316 (14)	0.33514 (18)	0.0564 (5)	

### Atomic displacement parameters (Å^2^)

**Table:**

	*U*^11^	*U*^22^	*U*^33^	*U*^12^	*U*^13^	*U*^23^
C3	0.0456 (15)	0.0474 (15)	0.0452 (15)	0.0024 (12)	0.0064 (12)	0.0044 (12)
C4	0.0541 (17)	0.0566 (18)	0.0479 (16)	−0.0064 (14)	0.0016 (13)	−0.0055 (13)
C5	0.0686 (19)	0.0408 (15)	0.0602 (17)	−0.0089 (14)	0.0021 (15)	−0.0062 (13)
C6	0.0570 (17)	0.0389 (14)	0.0523 (16)	0.0003 (12)	0.0043 (13)	0.0024 (12)
C1	0.0445 (14)	0.0380 (14)	0.0454 (14)	0.0009 (11)	0.0047 (11)	−0.0006 (11)
C2	0.0461 (15)	0.0383 (14)	0.0490 (15)	−0.0006 (12)	0.0064 (12)	−0.0027 (12)
C7	0.0510 (16)	0.0417 (15)	0.0537 (16)	0.0010 (13)	0.0032 (12)	0.0006 (13)
C8	0.0645 (19)	0.0666 (18)	0.0539 (17)	−0.0020 (15)	−0.0143 (14)	0.0017 (14)
N1	0.088 (2)	0.0614 (17)	0.0573 (16)	0.0015 (15)	−0.0116 (14)	0.0065 (14)
O2	0.1039 (18)	0.0617 (14)	0.0704 (14)	0.0079 (13)	−0.0123 (12)	0.0118 (11)
O3	0.130 (2)	0.0832 (17)	0.0741 (16)	−0.0058 (15)	−0.0405 (15)	0.0098 (13)
O1	0.0776 (14)	0.0355 (10)	0.0703 (13)	−0.0033 (9)	−0.0130 (10)	0.0026 (9)
O4	0.0805 (14)	0.0404 (11)	0.0720 (13)	−0.0043 (10)	−0.0188 (11)	−0.0055 (9)
O5	0.0603 (12)	0.0469 (11)	0.0532 (11)	−0.0018 (9)	−0.0089 (9)	0.0030 (9)

### Geometric parameters (Å, °)

**Table:**

C3—C4	1.380 (3)	C2—O1	1.334 (3)
C3—C2	1.396 (3)	C7—O4	1.215 (3)
C3—N1	1.457 (3)	C7—O5	1.323 (3)
C4—C5	1.369 (3)	C8—O5	1.455 (3)
C4—H4A	0.9300	C8—H8C	0.9600
C5—C6	1.371 (3)	C8—H8B	0.9600
C5—H5A	0.9300	C8—H8A	0.9600
C6—C1	1.383 (3)	N1—O2	1.200 (3)
C6—H6A	0.9300	N1—O3	1.201 (3)
C1—C2	1.408 (3)	O1—H1A	0.9600
C1—C7	1.470 (3)		
			
C4—C3—C2	121.4 (2)	O1—C2—C1	121.7 (2)
C4—C3—N1	117.0 (2)	C3—C2—C1	117.6 (2)
C2—C3—N1	121.6 (2)	O4—C7—O5	122.6 (2)
C5—C4—C3	120.3 (2)	O4—C7—C1	123.6 (2)
C5—C4—H4A	119.8	O5—C7—C1	113.8 (2)
C3—C4—H4A	119.8	O5—C8—H8C	109.5
C4—C5—C6	119.5 (2)	O5—C8—H8B	109.5
C4—C5—H5A	120.3	H8C—C8—H8B	109.5
C6—C5—H5A	120.3	O5—C8—H8A	109.5
C5—C6—C1	121.5 (2)	H8C—C8—H8A	109.5
C5—C6—H6A	119.2	H8B—C8—H8A	109.5
C1—C6—H6A	119.2	O2—N1—O3	121.4 (2)
C6—C1—C2	119.7 (2)	O2—N1—C3	120.2 (2)
C6—C1—C7	121.9 (2)	O3—N1—C3	118.3 (3)
C2—C1—C7	118.4 (2)	C2—O1—H1A	108.9
O1—C2—C3	120.7 (2)	C7—O5—C8	116.16 (19)

### Hydrogen-bond geometry (Å, °)

**Table:**

*D*—H···*A*	*D*—H	H···*A*	*D*···*A*	*D*—H···*A*
O1—H1A···O4	0.96	1.70	2.554 (2)	146
C4—H4A···O2^i^	0.93	2.57	3.321 (3)	138
C6—H6A···O4^ii^	0.93	2.49	3.336 (3)	151
C8—H8B···O1^ii^	0.96	2.59	3.305 (3)	131

Symmetry codes: (i) −*x*+1, *y*−1/2, −*z*−1/2; (ii) −*x*+2, *y*−1/2, −*z*+1/2.
